# Drug research and development opportunities in low- and middle-income countries: accelerating traditional medicine through systematic utilization and comprehensive synergy

**DOI:** 10.1186/s40249-022-00954-4

**Published:** 2022-03-07

**Authors:** Guangqi Liu, Yan Xie, Yinuo Sun, Kaixuan Zhang, Jiyan Ma, Yangmu Huang

**Affiliations:** 1 Department of Global Health, Peking University School of Public Health, 38 Xue Yuan Road, Haidian District, Beijing, 100191 China; 2 Institute for Global Health, Peking University, Beijing, China

**Keywords:** Traditional medicine, Research and development, Low- and middle-income countries

## Abstract

**Background:**

Though the utilization of traditional medicine has been proposed for modern drug research and development (R&D), limited research has discussed its feasible paths. In this commentary, we summarized key factors for new drug R&D under limited resources by reviewing China’s discovery of artemisinin, and raised suggestions to utilize traditional medicines in low- and middle-income countries (LMICs).

**Main text:**

We suggested that systematic utilization of traditional medicine, outstanding synergy of research units at all levels and timely information-sharing mechanism should be achieved to establish a comprehensive and efficient R&D system, especially under low-resource settings. In the case of artemisinin discovery, Chinese scientists integrated documented traditional medicine experiences and modern approaches to develop drug candidates timely. Due to limited R&D resources, China adopted a collaborative way, motivating nearly all domestic research units at different levels, to develop antimalarial products. Moreover, the excellent synergy among all units through efficient information-sharing mechanisms greatly avoided work repetition and accelerated the R&D process.

**Conclusion:**

Traditional medicines inspires drug discoveries in LMICs, while a comprehensive and efficient R&D system could accelerate its R&D process and save investment. The discovery of artemisinin in China gave a reliable pattern to promote sustainable development of traditional medicines and a good example to realize R&D of traditional medicine under low-resource settings.

## Background

Traditional medicines were developed on the basis of indigenous experiences from different cultures within a local environment [1]. It has benefited human health for thousands of years, and its contribution to health care was widely acknowledged, especially in low- and middle-income countries (LMICs) [2, 3]. In 2019, traditional Chinese medicine (TCM) was included into the International Classification of Diseases (ICD-11) system by the World Health Organization (WHO), demonstrating the increasing recognition and research interest of TCM worldwide [4]. Apart from direct contribution in local communities, traditional medicine has been utilized for new drug discovery by modern scientific techniques and methods [5]. Not only people worldwide could benefit from the research and development (R&D) of traditional medicine products, but also LMICs could consider the industrialization of traditional medicine products as an economic growth point and a sign of influence [6].

With the growing consensus about the tremendous potential of traditional medicine to advance human health, a systematic and adaptive R&D model should be established to guide the process in LMICs. Evidence showed that many LMICs, including Vietnam, India, Turkey, Mexico, Tanzania and so on, possessed abundant traditional medicine resources, but the R&D of traditional medicine in most countries were in early stage due to social and economic restrictions [7]. Some scientists argued that a more collaborative interaction among multi-level public and private sectors are needed to transform rich natural resources into products, and suggested that the research infrastructure should be improved to act as the drug development pipeline [8].

In China, traditional medicines have been valued by researchers from both public and private sectors and has inspired plenty modern drug discoveries, which set good examples for integrating modern sciences and traditional medicines [9]. Several new drugs developed from TCM have been modernized and contributed to a wide population range, such as the artemisinin for treating malaria [10]. Artemisinin-based combination therapy was acknowledged by WHO in the guidelines for the treatment of malaria (3rd edition) and has saved millions of lives worldwide [11]. The research and industrialization of artemisinin accumulated valuable experiences for developing other traditional medicines in LMICs.

The aim of this study was to raise suggestions to accelerate new drug R&D from traditional medicine under low-resource settings. This study took the successful discovery of artemisinin as an example and determine key factors to promote the vitality of traditional medicine in LMICs.

## Main text

Like other LMICs, China started new drug R&D under low-resource settings. China discovered artemisinin not only based on valuable experiential wisdom of TCM, but also relied on a government-led R&D pattern. Based on the accumulated experience from TCM and productive synergy among multiple departments, China discovered the amazing effectiveness of artemisinin as a novel cure against malaria. Three key factors towards success are still inspiring nowadays, including scientific utilization of traditional medicine, comprehensive synergy of all research units and efficient information-sharing mechanism.

### Targeting potential drugs from traditional medicine by modern methods

**Fig. 1:**
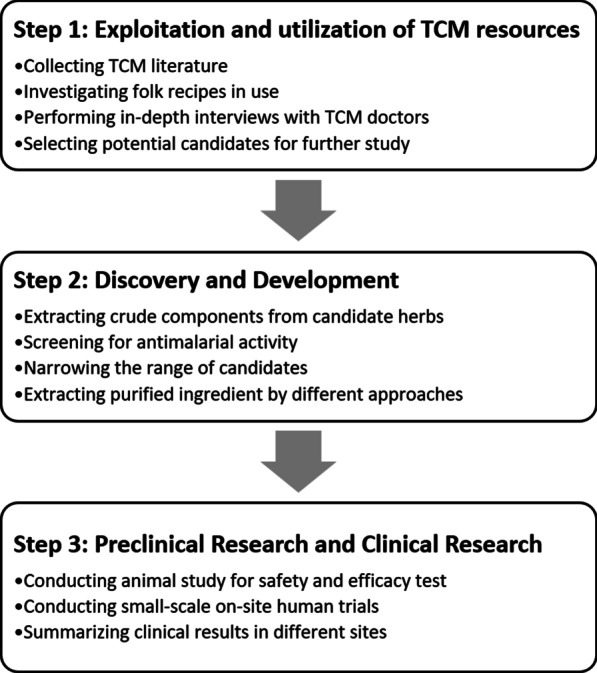
Exploration flow of antimalarial drugs on the basis of TCM. *TCM* Traditional Chinese medicine.

In pharmaceutical industry, the drug discovery process was acknowledged as expensive, risky and inefficient. Under the surveillance of national drug regulatory agency and WHO, all new drug R&D should stick to standard drug development and approval process, including preclinical studies and 3 phases of investigational clinical trials [12]. However, cases from China and other countries proved that traditional medicine could expedite the R&D process and save investment. In the case of artemisinin discovery, Chinese scientists quickly targeted drug candidates based on TCM resources and adhered to modern drug R&D procedures to develop drug candidates quickly (as shown in Fig. 1).

With profound knowledge of traditional medicine, Chinese scientists screened over 40,000 herbs and compounds from TCM books and collected folk remedies. After concluding the current evidence, 10 key herbs including Qinghao (Chinese name of *Artemisia annua* Linn, raw material of artemisinin) were selected for further research. Qinghao has been used for more than 2,000 years in China and its antimalarial effects was first recorded about 1,600 years ago in Ge Hong’s “A Handbook of Prescriptions for Emergencies” [13]. While extracting purified ingredient from Qinghao extraction, Youyou Tu was inspired by TCM literature and used low-temperature ethyl ether extraction instead of boiling extraction methods to protect the active ingredient. Also, animal experiments were used for safety and efficacy tests. The low-temperature ethyl ether extract of Qinghao showed 100% inhibition against mouse malaria, *Plasmodium berghei*.

Many LMICs were gradually aware of the value of traditional medicine. To effectively utilize traditional medicine, other LMICs could apply the exploration flow of artemisinin into their drug discovery process. It is worth noting that documented materials, folk practices and interviews from TCM doctors were equally valuable and they could provide evidence from different perspectives, including efficacy and safety.

### Establishing a cross-department and cross-region collaboration system

The inspirations from traditional medicine provided good foundation for new drug R&D process. However, the inadequate social and economic development of LMICs resulted in the shortage of funds and talents in related industries and greatly impeded the R&D process. A comprehensive collaboration system should be established to mobilize resources, while government should play a leading role in promoting synergy among all parties.

In the 1970s, China possessed extremely limited R&D resources and adopted a collaborative way to develop antimalarial products, which motivated nearly all domestic research units at different levels and departments. The national leadership group for malaria control and prevention drug research (set up on May 23, 1967, therefore referred to as National 523 Office) was established to take charge of research plan implementation and collaboration among regions and departments. The National 523 Office was composed of six sectors, including the State Scientific and Technological Commission, the General Logistics Department of Chinese People's Liberation Army, Science, Technology and Industry Commission for National Defense, Ministry of Health, Ministry of Chemical Industry, and the Chinese Academy of Sciences. Each sector would designate focal points to be responsible for communication with National 523 Offices and implementation of all tasks in their subordinate units in a timely manner. Dozens of research institutions were organized by National 523 Office and Provincial 523 offices, including scientific research centers, medical colleges, and pharmaceutical manufacturing facilities.

Traditional medicine was viewed as one of the research paths and a public private partnership was established to promote its development and industrialization. To overcome the challenge of producing artemisinin, the National 523 Office arranged for additional cooperation among institutions, medical colleges, and private enterprises [14]. In the industry-university-research cooperation chain, universities conducted clinical trials and provided convincing observation data about artemisinin’s effects against falciparum malaria; research institutes were engaged in structural analysis of artemisinin, and proposed that its possible structural formula contained lactone peroxide; while pharmaceutical manufacturing facilities carried out investigations on raw materials and quality standards, etc.

During the process, government departments collected research information from all sectors and served the whole industry chain by providing clear division and guidance. This government-led and mission driven R&D pattern could be used to establish a comprehensive and efficient R&D system for resource limited settings. This collaboration system has also been seen during the COVID-19 period domestically and globally to accelerate the R&D process.

### Promoting synergy through efficient information-sharing mechanisms

A productive collaboration system would not only rely on the commitment of government, but also build on efficient information-sharing mechanisms to promote synergy. Cross-region collaboration needed regular communications to avoid work repetition and share recent breakthroughs. China’s research on artemisinin set a good example in the 1970s. After Youyou Tu reported the discovery of Qinghao, China’s National 523 Office and professional groups attached great importance and promoted the information sharing among all research units and adjusted research directions. The information exchange among provinces ensured that all research centers progressed based on the latest breakthroughs and the synergy raised the efficiency of all researches.

Nowadays, with the development of communication technologies, information and progress could be exchanged online instead of by offline meetings, which was much more time-saving and convenient. With the help of emerging communication tools, LMICs could establish a more effective information-sharing mechanisms to promote R&D collaborations.

## Conclusions

The discovery of artemisinin not only contributed to the reduction of malaria mortality globally, but also set an example for R&D of traditional medicine under low-resource settings in LMICs. Through collecting related records, screening promising candidates and identifying active ingredients, researchers could take advantage of ancestors’ wisdom in traditional medicine and explore new drugs. However, new drug development required multidisciplinary talents and massive financial support. Under low-resource conditions, China achieved outstanding innovative development of antimalarial drugs, which was greatly attributed to systematic utilization of traditional medicine, outstanding synergy of research units at all levels and timely information-sharing mechanism. To promote comprehensive and sustainable development of traditional medicine, LMICs should identify weaknesses in research resources and develop adaptive drug R&D pattern based on China’s experience of discovering artemisinin. The following recommendations should be valued when concrete executive strategies on drug R&D were generated: strengthening the utilization of traditional medicine by systematic review and in-depth interview; building R&D pipeline for targeted drug candidate by modern methods; exerting the leading role of government by integrating multidisciplinary research units and establishing information-sharing mechanism.

## Data Availability

Not applicable.

## Abbreviations

WHO: World Health Organization
R&D: Research and development
LMICs: Low- and middle-income countries
TCM: Traditional Chinese medicine
